# Osteoblast-Like Cell Behavior on Porous Scaffolds Based on Poly(styrene) Fibers

**DOI:** 10.1155/2014/609319

**Published:** 2014-06-19

**Authors:** Andrada Serafim, Romain Mallet, Florence Pascaretti-Grizon, Izabela-Cristina Stancu, Daniel Chappard

**Affiliations:** ^1^Advanced Polymer Materials Group, University Politehnica of Bucharest, 149 Calea Victoriei, Sector 1, 010072 Bucharest, Romania; ^2^GEROM Groupe Etudes Remodelage Osseux et bioMatériaux-LHEA, IRIS-IBS Institut de Biologie en Santé, LUNAM Université, 49933 Angers Cedex, France

## Abstract

Scaffolds of nonresorbable biomaterials can represent an interesting alternative for replacing large bone defects in some particular clinical cases with massive bone loss. Poly(styrene) microfibers were prepared by a dry spinning method. They were partially melted to provide 3D porous scaffolds. The quality of the material was assessed by Raman spectroscopy. Surface roughness was determined by atomic force microscopy and vertical interference microscopy. Saos-2 osteoblast-like cells were seeded on the surface of the fibers and left to proliferate. Cell morphology, evaluated by scanning electron microscopy, revealed that they can spread and elongate on the rough microfiber surface. Porous 3D scaffolds made of nonresorbable poly(styrene) fibers are cytocompatible biomaterials mimicking allogenic bone trabeculae and allowing the growth and development of osteoblast-like cells *in vitro*.

## 1. Introduction

The increasing frequencies of traumatic and pathologic bone defects, as well as the skeletal problems due to osteoporosis and bone degeneration in aging population, request a societal need for improved therapeutic products. The need for biomimetic scaffold materials as alternative to bone auto- or allografts is well recognized [1]. Consequently, the investigation of artificial materials for bone repair remains a constant key concern in the field of biomaterials research for clinical applications. It is widely accepted that three-dimensional porous constructs are needed to lodge bone cells and to provide the template for tissue formation and development, including angiogenesis. Synthetic or natural ceramics are either too brittle to be used in weight-bearing bones (e.g., *β*-Tri calcium phosphate) or massive and impossible to resorb by bone remodeling (e.g., coral or hydroxyapatite blocks). Biodegradable biocompatible polymers have been proposed to fill small bone defects. Clinical situations exist where large bone defects cannot be replaced by such resorbable materials which also present important drawbacks (e.g., polylactic or polyglycolic acid) [2, 3]. Large bone flaps of the skull are done in neurosurgery for the treatment of tumors or large pelvic amputations occurring in the case of sarcoma or metastasis. Reconstruction with a variety of nonresorbable materials has been proposed (tantalum or titanium plates, PMMA plates prepared with bone cement…) but none has been found satisfactory [4]. For example, PMMA is biotolerated and always encapsulated by a thin layer of fibrosis. Unlike biodegradable macromolecules, nonbiodegradable and biocompatible polymers such as polyhydroxyethyl methacrylate and poly(styrene) could represent interesting solutions to generate permanent scaffolds supporting bone regeneration in case of extensive bone losses. We have investigated polyhydroxyethyl methacrylate as an interesting synthetic hydrogel for bone biomaterials [5–8]. However, its use in load bearing applications may be limited by insufficient mechanical strength in hydrated form. Therefore, we considered the potential of poly(styrene), the most widely used polymer in tissue culture, for such applications. To ensure bone osseointegration after grafting, we decided to prepare poly(styrene) scaffolds with an interconnected porosity allowing the colonization by bone cells and vascular sprouts. Porosity of a bone substitute plays a critical role due to the fact that the bone is a highly vascularized tissue, and therefore the success of an implant is correlated with its capacity to induce angiogenesis and insure cell migration and proliferation [9, 10]. A variety of techniques to generate porous materials are available and among them, the self-assembling of fibers provides interconnected porosity with geometry strictly dependent on the dimension and morphological characteristics of the fibers. Recently, scaffolds for tissue engineering based on electrospun poly(styrene) nanofibers have attracted increased attention [11–14]. Electrospinning provides nanostructured porous membranes and surfaces that can be successfully used to promote cell adherence, spreading, and proliferation due to their resemblance with the nanostructured microenvironment osteoblasts that are accustomed within hard tissues. However, this technique is not available to produce thick scaffolds and size of the nanofibers is in the range of a single collagen fibril. On the other hand, the resulting porosity is too low to provide colonization of thick scaffolds with osteoblasts. Such limitations can be overcome through the entanglement of larger polymer fibers. In addition, another factor which has a crucial influence on the success of an implant is represented by the interaction between the surface roughness of the implanted material and the cells from the native tissue [15, 16]. Osteoblast cells are anchorage-dependent and require a rough surface to adhere [17–22]. Therefore, in the present study, we report the potential of rough poly(styrene) microfibers to generate porous scaffolds for bone regeneration. The morphology of the fibers was investigated through atomic force microscopy, vertical interference microscopy, and scanning electron microscopy. On the long term, poly(styrene) fibers aim to be used to develop thick porous scaffolds for bone regeneration. Therefore, the response of osteoblasts to such materials has also been assessed.

## 2. Materials and Methods

### 2.1. Preparation of Poly(styrene) Fibers

Poly(styrene) with an average *M*_*w*_ ≈ 192000 was purchased from Aldrich Chemical Company.* N,N*-dimethylformamide (DMF) (99.5%) was purchased from Merck and methanol (99.5%) from Fisher Chemical. All reagents were used as such. A few drops of methanol were added to the solution of poly(styrene) in DMF (7% w/v) until a soft gummy precipitate is formed. Dry spinning was used to obtain thin fibers of poly(styrene), in air, at room temperature. Then, the fibers were washed several times with ethanol and water, to remove residual DMF and dried in the oven at 37°C.

### 2.2. Preparation of Porous Poly(styrene) Scaffolds

**Scheme 1:**
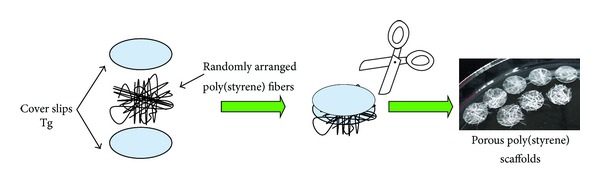
Preparation of porous poly(styrene) scaffolds through randomly assembling of fibers.

Sintering of the polymer at its glass transition temperature was performed between two cover slips, to generate randomly assembled 3D scaffolds, as depicted in Scheme 1. Sterilization occurred through exposure to UV radiation at a wavelength of 260 nm, at room temperature, overnight.

### 2.3. Characterization of Poly(styrene) Fibers

#### 2.3.1. Raman Spectroscopy

Raman spectra of the fibers were recorded on a Senterra Raman microscope. The excitation laser wavelength was 532 nm using a laser power level of about 25 mW. The Raman wavenumber range was between 500 and 3200 cm^−1^. The estimated Raman resolution was 3–5 cm^−1^. For the data collection, the exposure time was of 10 seconds.

#### 2.3.2. Atomic Force Microscopy (AFM)

The surface roughness of poly(styrene) fibers was observed by atomic force microscopy (AFM) using a CP-Research AFM from ThermoMicroscopes, Bruker. The apparatus was operated in contact mode, thus permitting a tight contact between the tip and the sample. Plotting the deflection of the cantilever against its position resulted in a topographic image of the surface.

#### 2.3.3. Vertical Interference Microscopy

Optical interferometric measurements were done using a Wyko NT 9100 optical profiling system (Bruker AXS, Champ sur Marne, France). The microscope is based on light interferometry and operates as a noncontact optical profiler in vertical scanning interferometry mode to produce 3D topography maps of the sample surface. Briefly, a white light source is emitted by conventional light source and is split into two beams which pass through a Mirau's interferometric objective. The incident beams are reflected from the reference mirror and the sample surface, respectively. The light reflected from this mirror combines with the light reflected from the sample to produce interference fringes (known as interferogram) where the best-contrast fringe occurs at best focus. The light and dark fringes are used in combination with the wavelength of the light to determine height difference between each fringe. A piezoelectric stage moves the sample vertically with a nanometer precision, which produces phase shifts in the interferogram. Interferograms were digitized using a CCD camera and data were analyzed to produce a topographic surface map. The software Vision (release 4.10, Wyco) was used to acquire the data.

#### 2.3.4. Scanning Electron Microscopy (SEM)

SEM was used to investigate the morphological features of the obtained polymer fibers. Samples were coated with a thin layer of gold prior to analysis, and images up to a magnitude of 10000X were recorded. The study was performed using a JEOL JSM-6301F SEM equipped with a conical FE electron gun with a resolution of 1.5 nm. Images were registered with an accelerated voltage of 3 kV at a working distance of 15 mm.

An environmental Zeiss Evo LS10 SEM was used to image the cell distribution on the poly(styrene) 3D scaffolds, after cell culture. Images were recorded in the back scattered mode, at a working distance of 9 mm with an accelerated voltage of 7 kV. The SEM is equipped with a tungsten gun and has a resolution of 3.0 nm at 15 kV. Prior to analysis, the samples were covered by sputtering with a 10 nm layer of carbon with a MED 020 (Bal-Tec, Balzers, Lichtenstein).

### 2.4. Cell Culture

Sterilized poly(styrene) scaffolds were transferred into 24 well plates containing Dulbecco's Modified Eagle's Medium (DMEM) enriched with fetal calf serum 10%, penicillin (100 UI/mL), and streptomycin (100 mg/mL). Samples were equilibrated in this medium for 1 h prior to cell culture. The Saos-2 osteoblast-like cells (issued from a rat osteosarcoma) were seeded at a concentration of 10^5^ cells/mL on the samples for 24, 48, 72, and 120 hours. Medium was refreshed every two days. During cell culture, samples were kept in a humidified incubator under 5% CO_2_. Experiments were made in triplicate. Glass cover slips were used as control samples.

At the end of each time point, the medium was discarded and samples were rinsed with phosphate buffer 0.1 M, pH 7.4, and fixed with 2.5% (w/v) glutaraldehyde solution in phosphate buffer for 24 h. Then, samples were rinsed with phosphate buffer, postfixed with osmium tetroxide for 45 minutes, and rinsed with distilled water. Then, the samples were dehydrated with a gradient of ethanol and desiccated with hexamethyldisilazane before SEM analyses. SEM images were processed using Amira 5.2.2 (Visage Imaging). Three randomly selected areas were analyzed. The ratio between the surface of cells covered fibers and the surface of noncovered fibers was used to quantify cells proliferation.

## 3. Results and Discussion

**Figure 1:**
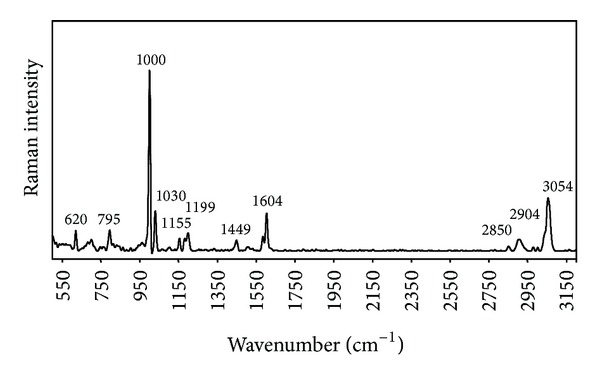
Raman spectrum recorded for poly(styrene) fibers obtained through dry spinning.

In order to verify the thoroughness of the washing process, the fibers were analyzed through Raman spectroscopy. There were no significant differences between the recorded spectrum (Figure 1) and the data recorded in the literature [23]; this confirmed that all the solvent has been removed.

**Figure 2:**
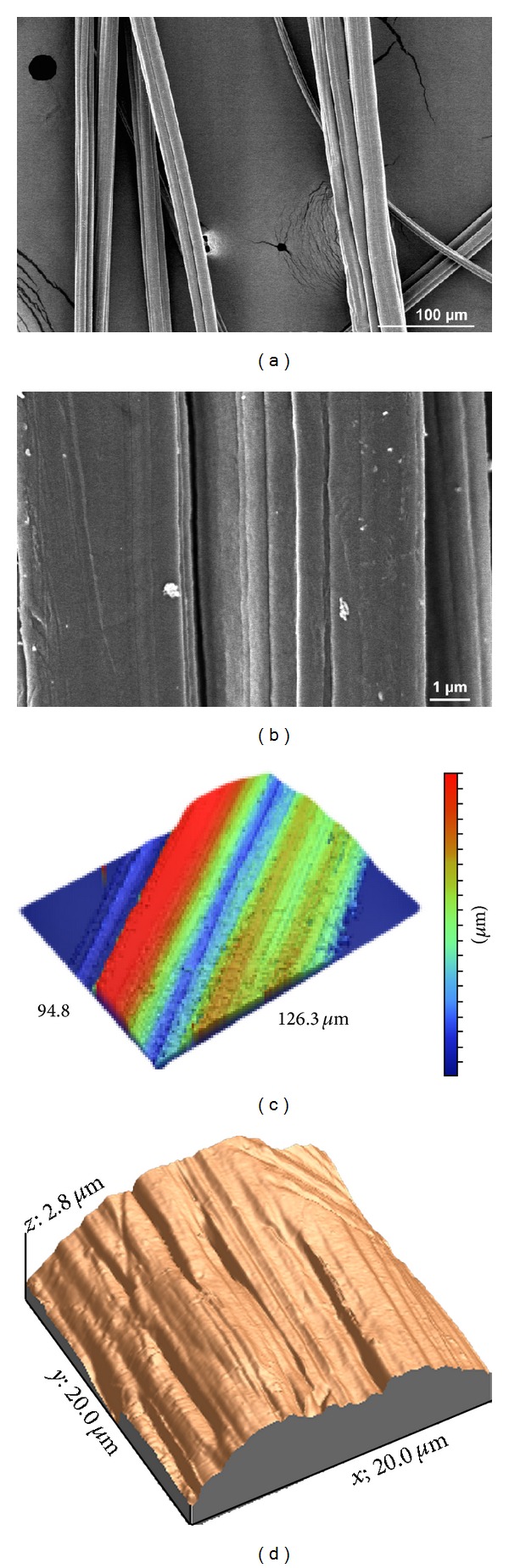
Morphology of the poly(styrene) fibers. (a), (b) SEM images at different magnifications ((a) 250x, (b) 10.000x); (c) microtopography obtained with vertical interference microscopy; and (d) surface topography observed through AFM.

The obtained fibers were further imaged to establish their morphological characteristics. Their diameter ranged from 5 to 50 *μ*m in thickness, similar to small bone trabeculae. The general morphology of the fibers was analyzed through SEM and surface topography was further investigated through AFM and vertical interference microscopy (Figure 2). SEM images revealed that the fibers were bead-free and nonporous and present microchannels orientated along the longest axis of the fiber. Microroughness was evidenced at the surface of the fibers. The mean roughness value (Ra), as estimated through vertical interference microscopy, was 5.32 *μ*m. This microscopy can analyze larger surfaces than AFM and provide similar quantitative results. It is nondestructive and well adapted to the study of bone and biomaterials [24–26].

**Figure 3:**
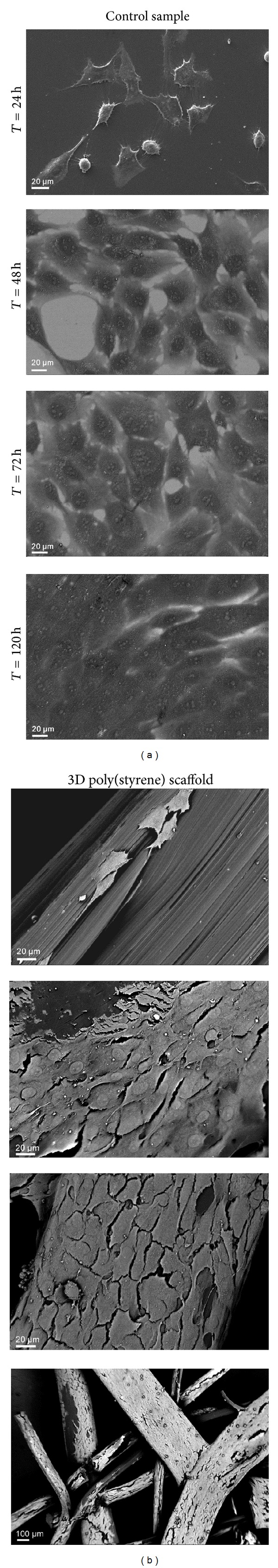
SEM micrographs showing osteoblasts adhered on cover slips (a) and poly(styrene) fibers (b) at various time points.

Cell-scaffold interaction and the osteogenic potential of the porous poly(styrene) scaffolds were assessed through cell cultures. The SEM images registered on the scaffolds showed that cells adhere on the fiber surface in less than 24 hours after seeding. Compared with the control sample (i.e., the glass cover slip), the cells attached on the poly(styrene) scaffolds are elongated and oriented along the fiber (Figure 3). Very similar aspects were described by our group when studying the effect of purified bone allografts. In culture, osteoblasts spread and elongate along the surface microtopography of the collagenic surface of allografts [25]. When aggressive purification processes are used to clean bones, the cells do not recognize the topography of the surface and proliferation rate is markedly reduced [27].

**Figure 4:**
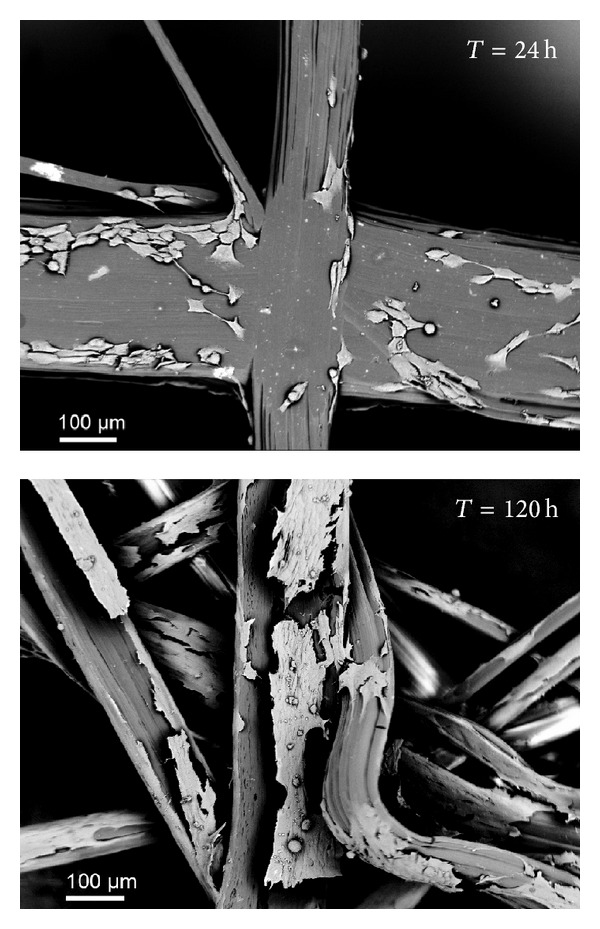
SEM (backscattered electron mode) images of Saos-2 cells on poly(styrene) fibers; magnification 400x.

Cells orientation along the poly(styrene) fibers was maintained while they proliferate, which indicates their osteoconductive properties (Figure 4). The cells have the tendency to elongate and spread along the fibers. A small amount of cells stretched across multiple points where multiple fibers converge.

**Figure 5:**
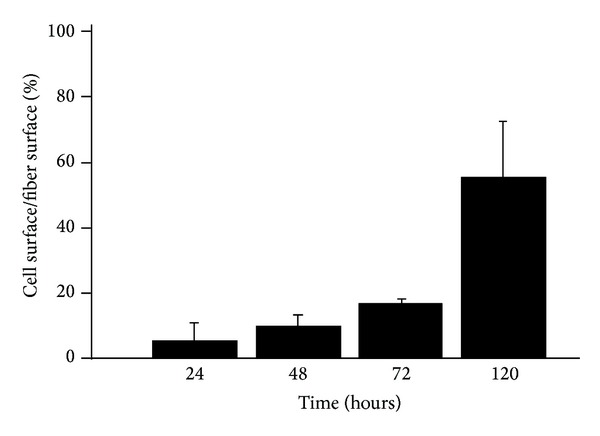
Saos-2 cells proliferation on 3D poly(styrene) scaffolds; error bars show standard deviation.

Cell proliferation was studied as a function of the culture time and results are presented in Figure 5. Doubling the seeding time resulted in approximately 7-fold increase of cell number. Further, increasing the seeding time resulted in an increased number of cells on the studied scaffolds.

## 4. Conclusions

The poly(styrene) fibers investigated in this study represent appealing substrates for the development of thick porous scaffolds for bone regeneration. In addition to porosity, such scaffolds present a suitable surface roughness that was proven to be efficient in stimulating osteoblasts adherence and proliferation [20]. Saos-2 cells were able to populate the entire surface of the porous scaffold after 120 hours. Additionally, Saos-2 cells were oriented along the longitudinal axis of the microgroves present on the fibers. Therefore, it is considered of paramount importance to develop such complex structures containing a rigid polymer framework in low amount to further assist the bone regeneration processes even on longer time. The present approach does not underestimate the potential of biodegradable polymers or ceramics in bone regeneration but in some clinical circumstances such as bone carcinologic surgery, the dogma of using resorbable materials that last for many years and has a tough life can be faulted. It offers a different perspective by investigating a new strategy mainly based on nonresorbable polymers in large bone defects where osteoconduction needs a solid scaffold. Other nonresorbable polymers (Goretex membranes, PHEMA coated with calcium hydroxide) have also been proposed successfully [28–30]. In vivo implantation of poly(styrene) scaffolds is now under study in our laboratory.
